# Extracellular vesicles derived from ascitic fluid enhance growth and migration of ovarian cancer cells

**DOI:** 10.1038/s41598-021-88163-1

**Published:** 2021-04-28

**Authors:** Aparna Mitra, Kyoko Yoshida-Court, Travis N. Solley, Megan Mikkelson, Chi Lam Au Yeung, Alpa Nick, Karen Lu, Ann H. Klopp

**Affiliations:** 1 Department of Radiation Oncology, The University of Texas MD Anderson Cancer Center, 1515 Holcombe Boulevard, Houston, TX 77030 USA; 2 Department of Gynecologic Oncology and Reproductive Medicine, The University of Texas MD Anderson Cancer Center, Houston, TX 77030 USA; 3 Tennessee Oncology, Nashville, TN 37203 USA

**Keywords:** Cancer, Medical research

## Abstract

Ovarian cancer is associated with a high mortality rate due to diagnosis at advanced stages. Dissemination often occurs intraperitoneally within the ascites fluid. The microenvironment can support dissemination through several mechanisms. One potential ascites factor which may mediate dissemination are EVs or extracellular vesicles that can carry information in the form of miRNAs, proteins, lipids, and act as mediators of cellular communication. We present our observations on EVs isolated from ascitic supernatants from patients diagnosed with high grade serous ovarian carcinoma in augmenting motility, growth, and migration towards omental fat. MicroRNA profiling of EVs from malignant ascitic supernatant demonstrates high expression of miR 200c-3p, miR18a-5p, miR1246, and miR1290 and low expression of miR 100- 5p as compared to EVs isolated from benign ascitic supernatant. The migration of ovarian cancer spheroids towards omental fat is enhanced in the presence of malignant ascitic EVs. Gene expression of these cells showed increased expression of ZBED2, ZBTB20, ABCC3, UHMK1, and low expression of Transgelin and MARCKS. We present evidence that ovarian ascitic EVs increase the growth of ovarian cancer spheroids through miRNAs.

## Introduction

Patients with ovarian cancer who develop chemoresistant disease often develop malignant ascites consisting of a heterogeneous mixture of cells, including tumor cells, stromal cells like fibroblasts, adipose-derived stromal cells, bone marrow stem cells, and acellular components like cytokines, metabolites, proteins, and extracellular vesicles (EVs)^1^. Tumor spheroids form within the ascites and can initiate metastasis within the visceral adipose tissue^2, 3^. Adhesion of cancer spheroids is facilitated by factors within the ascites. The ascitic fluid is composed of constituents such as lipids, proteins, and EVs^4, 5^ which includes exosomes, microvesicles, or apoptotic blebs^6, 7^. The critical factors in malignant ascites that support this spheroid-mediated process of omental metastasis are poorly understood. We hypothesized the EVs may be one critical mediator of omental metastasis. To investigate this, we characterized EVs isolated from whole ascites of ovarian cancer patients. We identified exosomal derived miRNAs, which mediated omental metastasis in an ex-vivo model. We describe our findings showing the role of ascitic fluid derived EVs in augmenting spheroidal growth of ovarian cancer cells via miRNAs and increasing cancer cell migration to the omentum. Notably, we show reduced expression of miR100, miR 125b, and increased expression of miR200a/b/c, miR1290, and miR1246 in EVs derived from ascitic fluid of patients diagnosed with high grade serous ovarian cancer as compared to EVs isolated from ascites of patients without cancer (Table 1).

**Table 1:**
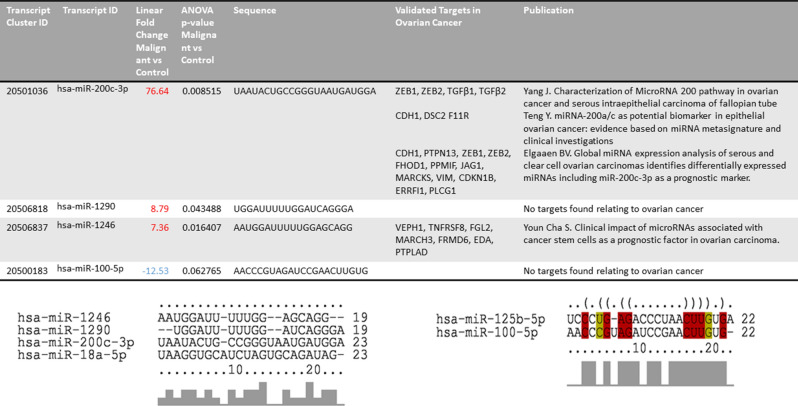
Micro-RNAs and their predicted targets in Extracellular vesicles isolated from ascitic supernatant.

## Results

### Characterization of EVs

**Figure 1:**
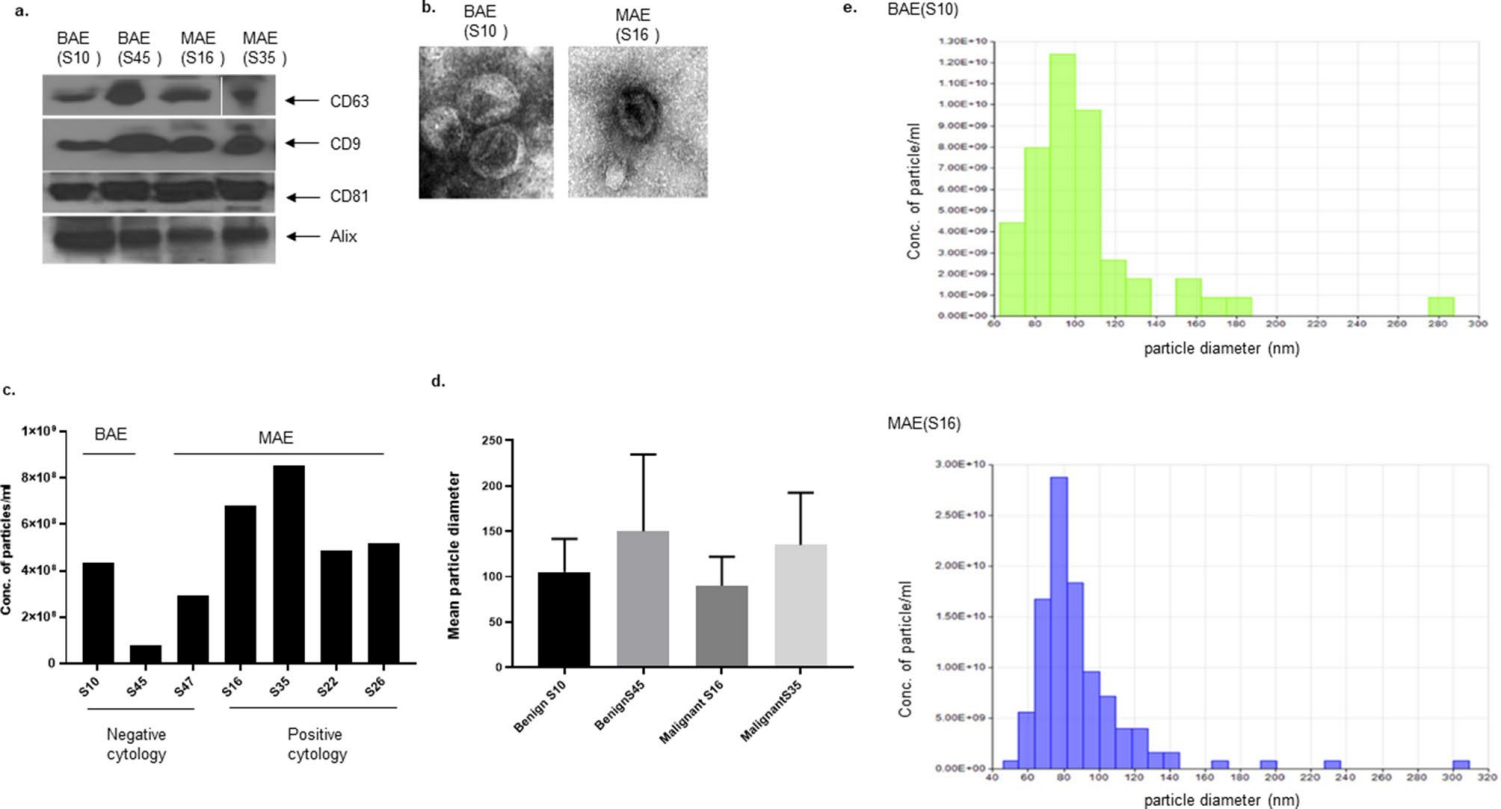
Characterization of exosomes. Expression of exosome specific markers (CD63, CD81, CD9, and Alix) in exosome derived proteins by western blot. Exosomes were either isolated from Benign tumor associated (BAE) or malignant tumor associated (MAE) ascitic supernatant (**a**). Representative TEM images of exosomes isolated from benign and malignant tumor associated ascitic supernatants (**b**). Mean exosomal particle diameter in benign and malignant tumor associated ascitic supernatants (**d**). The concentration of particles per ml of ascites derived supernatants measured by qNano (**c**,**e**).

EVs were isolated from ascitic supernatant from patients diagnosed with high grade serous ovarian cancer (MAE) or benign disease (BAE). BAE was collected from two patients taken for surgical staging of suspected ovarian cancer who were instead diagnosed with benign disease, Meig's syndrome, or borderline tumor (Table 2, study no. S10, no. S45). MAE and BAE expressed characteristic exosomal markers CD63, CD9, CD81, and Alix (Fig. 1a). Electron micrographs demonstrated that MAE and BAE were comparable in size and shape (Fig. 1b). qNano analysis of EVs isolated from an equal amount of supernatant collected from patient ascites demonstrated a higher number of EVs in malignant ascitic supernatant than from benign ascitic supernatant (Fig. 1c). The mean diameter of EVs was not significantly different (Fig. d). Figure 1e shows representative diameters of benign ascites, and malignant ascites derived EVs. The majority of the vesicles are 40–140 nm diameter range.

**Table 2:** Patient characteristics.

	Study no.	Ascites malignancy result; 0 = No; 1 = Yes	Days from ascites collected to the first surgery	Age at diagnosis	Ethnicity	BMI	ca125 of date ascites extracted	Original tumor location; 1 = Ovary; 2 = peritoneum	Stage; 8 = IIIc; 9 = IV; 10 = unstaged	Histology; 1 = serous/papillary serous; 3 = endometrioid	Grade; 1= High; 2= Low	Chemothrapy; 0 = No; 1 = Yes	Reductive surgery; 1 = Yes; No = 0	Platinum sunsitivity; 1 = sensitive; 2 = resistance	Survival Status; 0 = alive; 1 = dead
Control	45	0	0	51	Black	52	793.5	1	10	3	n/a	0	1	n/a	0
	10	0	0	73	White	23	n/a	n/a	10	n/a	n/a	n/a	1	n/a	0
HGSOVCA (PS)	35	1	9	57	White	43	820.6	1	8	1	1	1	1	1	0
	48	1	0	46	White	26	11837	1	9	1	1	1	1	1	0
	49	1	0	59	White	19	551.3	1	9	1	1	1	1	1	0
HGSOVCA (PR)	16	1	0	62	White	28	1116	1,2	8	1	1	1	1	2	1
	47	0	0	59	White	27	286.3	1	8	1	1	1	1	2	0

### EVs isolated from the supernatant of patient-derived ascites increased the growth of ovarian cancer spheroids

**Figure 2:**
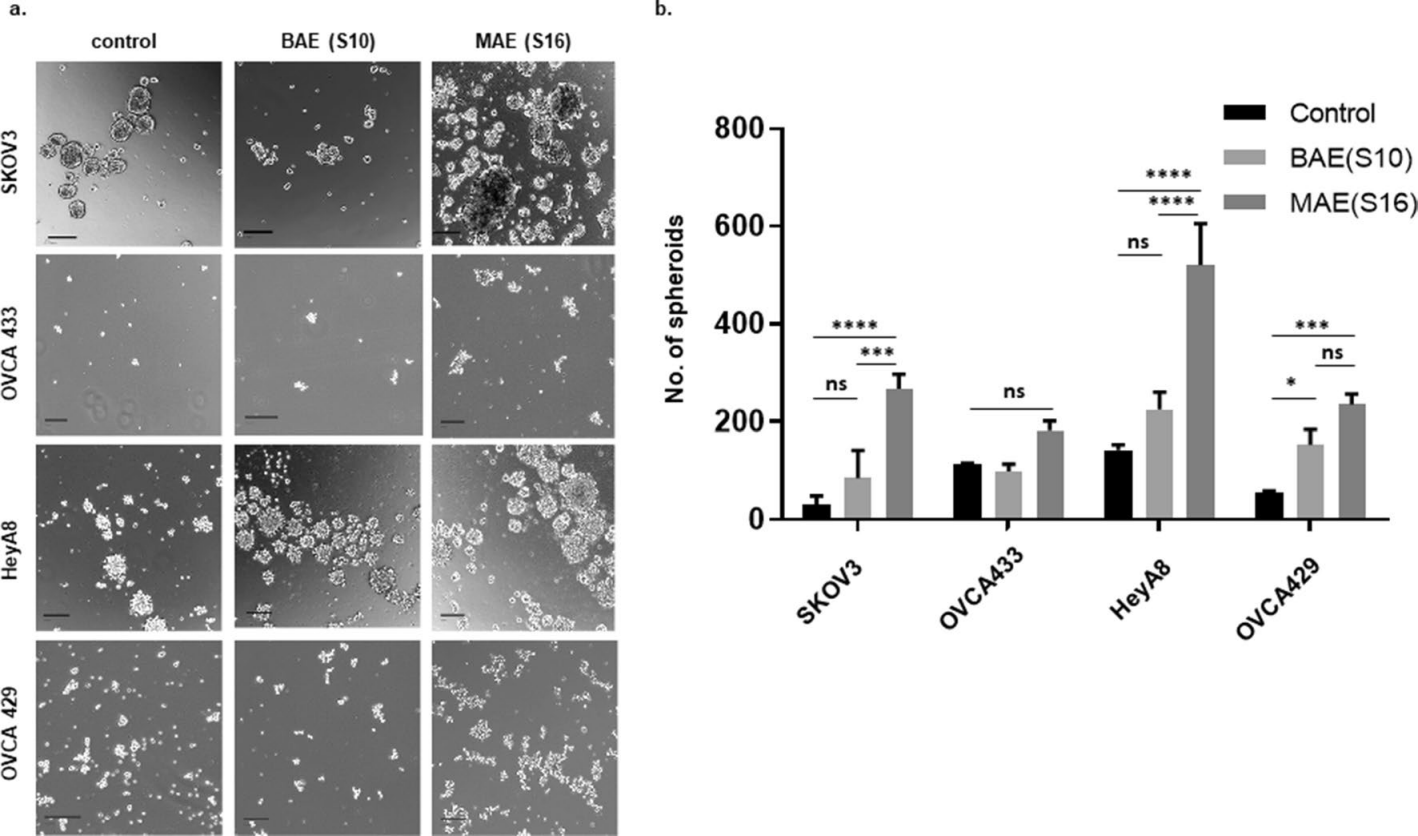
Exosomes isolated from benign and malignant tumor associated ascitic supernatants increase the growth of ovarian cancer spheroids. Ovarian cancer cells SKOV3, OVCA433 HeyA8, and OVCA429 were grown in low attachment plates as spheroids as control (without exosomes), as BAE (grown with 250ug/ml exosomes from benign ascitic supernatant) or as MAE (grown with 250ug/ml exosomes from malignant ascitic supernatant). Spheroids were photographed (**a**) after seven days and quantitated using an Oxford-Optronix Gelcount automated cell counting instrument (Sarasota Fl.) and represented as a bar graph (**b**).

Ovarian cancer cells HeyA8, SKOV3, OVCA433, and OVCA429, were grown as spheroid cultures in low attachment plates with 250ug/ml EVs for seven days (Fig. 2a). Treatment with malignant ascitic supernatant derived EVs (MAE) increased spheroid growth relative to spheroids grown in the presence of benign ascitic supernatant derived EVs (BAE) in SKOV3 (control vs. MAE p = 0.0001; BAE vs. MAE p = 0.0006), HeyA8(control vs. MAE p = 0.001; BAE vs. MAE p = 0.006), and OVCA429 cells (control vs. BAE p = 0.04; control vs. MAE p = 0.0007) (Fig. 2b).

### Ascitic supernatant-derived EVs increased in vitro migration, colony formation, and wound healing capacity, and ex vivo migration to omentum of ovarian cancer cells

**Figure 3:**
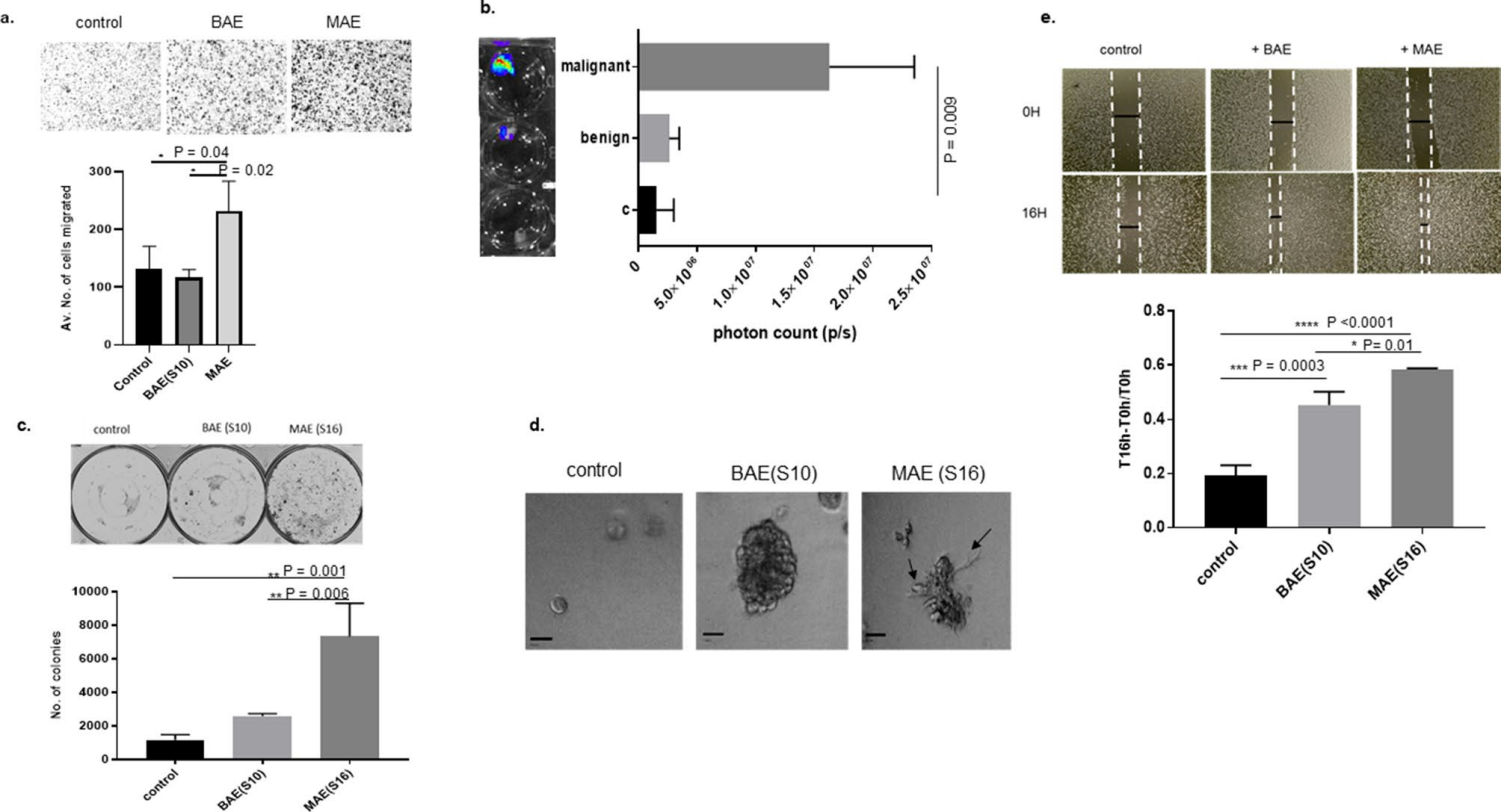
Malignant supernatant derived ascitic exosomes increase in vitro and ex-vivo migration, colony formation, and growth in a 3D matrix. Ovarian cancer cells with MAE or BAE were allowed to migrate through 8 μm polyethylene terephthalate filters along with control cells (without exosomes). Cells in the presence of MAE showed enhanced migratory capabilities as compared to those grown with BAE or controls (**a**). Luciferase labeled ovarian cancer SKOV3 cells were also allowed to grow with pieces of human omental tissue treated with 250 µg exosomes along with controls (untreated omentum) for 5 days. The number of migrated labeled cells was assayed using an IVIS imager (**b**). Ovarian cancer cells treated with BAE or MAE were allowed to form colonies in a six well plate. After a week, the colonies were stained with 0.05% crystal violet, and the plate was scanned to detect colonies using a Gelcount cell counting instrument. The number of colonies in the cells grown with malignant ascites appears to be significantly more than the benign exosome treated ones or controls grew without exosomes (**c**). SKOV3 cells grown in the presence of exosomes derived from benign ascites or malignant ascites were allowed to grow as 3D Cultures in 3D Matrix Basement Membrane Extract. Arrows indicate invasive growth of cells grown along with malignant ascites derived exosomes (MAE) in the 3D matrix. In contrast, the control cells or cells grown along with exosomes from benign ascites do not show any features of stellate growth (**d**). Scratch assay showed increased motility of cells grown in the presence of exosomes derived from malignant ascites (MAE) as compared to those grown in the presence of benign ascites (BAE) (**e**).

Ovarian cancer cells treated with equivalent numbers of MAE or BAE were allowed to migrate through 8 μm polyethylene terephthalate filters along with untreated controls. Cells in the presence of MAE showed enhanced migratory capabilities as compared to those grown with BAE (p = 0.02) or controls (p = 0.04) (Fig. 3a). The capacity to initiate omental metastasis was evaluated using human omentum incubated ex-vivo with SKOV3(rLuc) cells with and without EVs. The number of migrated labeled cells was assayed using an IVIS imager (Fig. 3b). There was a significant increase in cell migration towards omentum treated with MAE (p = 0.009).

Ovarian cancer cells treated with BAE or MAE were allowed to form colonies in a six-well plate. The number of colonies in the cells grown with malignant ascites was significantly higher than spheroids incubated with BAE (p = 0.006) or control untreated spheroids (p = 0.001) (Fig. 3c). SKOV3 cells grown in the presence of EVs derived from benign ascites or malignant ascites developed features of invasion with stellate growth when grown as 3D Cultures in 3D Matrix Basement Membrane Extract. Arrows in Fig. 3d indicate invasive growth of cells grown along with MAE in the 3D matrix, whereas the control cells or cells grown along with EVs from benign ascites do not show any features of stellate growth (Fig. 3d). Scratch assays demonstrated increased motility in cells grown in the presence of MAE as compared to BAE (p = 0.01) and control (p < 0.0001) (Fig. 3e).

### Exosomal miRNA alter growth and etoposide sensitivity of ovarian cancer spheroids

**Figure 4:**
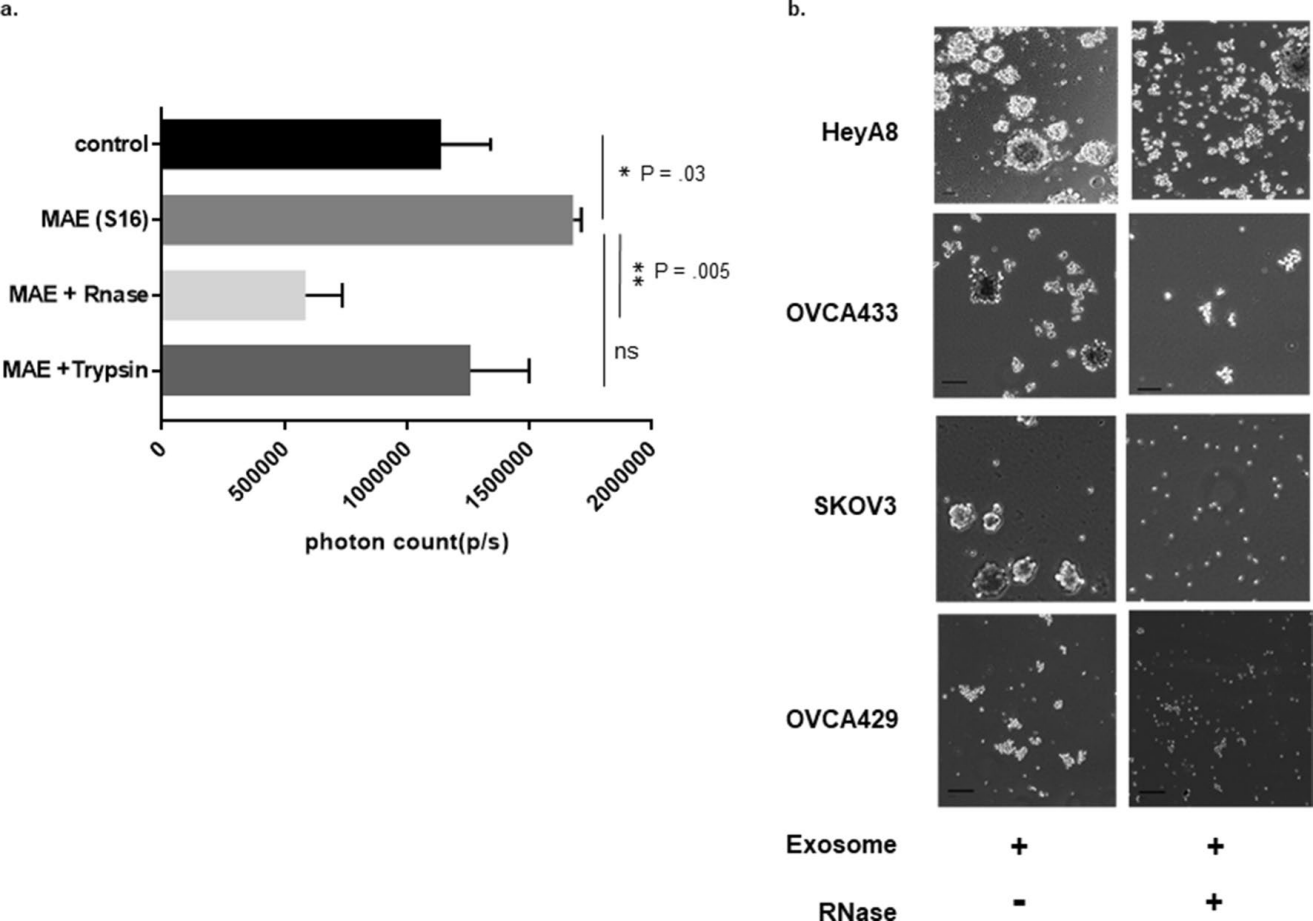
Increased growth of ovarian cancer spheroids is mediated by exosomal miRNA. Ovarian cancer cells HeyA8, SKOV3, OVCA433, or OVCA429, were grown with malignant exosomes treated with RNase along with non-treated malignant exosomes for seven days and assayed for spheroid forming capacities. We found that RNase treatment decreased both size (**b**) and a number of spheroids (**a**,**b**), indicating a possible role of exosomal RNA.

To determine if the observed effects of ascites supernatant were due to miRNA or proteins, luciferase labeled ovarian cancer SKOV3 cells were also allowed to migrate towards pieces of human omental tissue treated with 250 μg of MAE alone, or MAE treated with RNase (final concentration 0.5 mg/ml; ThermoFischer Scientific, Waltham, MA) for 20 min or Trypsin. The number of migrated labeled cells was assayed using an IVIS imager for five days (Fig. 4a). MAE increased migration towards the omentum. This effect was abrogated by RNase treatment of EVs but not Trypsin suggesting that extra or perivesicular RNAs were responsible for mediating this effect (p = 0.005). Ovarian cancer cells HeyA8, SKOV3, OVCA433, or OVCA429 were grown with malignant EVs treated with RNase along with non-treated malignant EVs for seven days and assayed for spheroid forming capacities. We found that RNase treatment decreased both size and number of spheroids indicating ascites supernatant effects on spheroid formation were mediated by exosomal RNA (Fig. 4b).

Ovarian cancer cells with EVs derived from both benign and malignant ascites were then treated with taxol, carboplatin, and etoposide for 72 h. MTT assays were done to assay for survival. Cells treated with EVs from malignant ascites were resistant to etoposide treatment, but EV treatment did not alter carboplatin or paclitaxel sensitivity (Supplementary Figure S4).

### miRNA profiling of exosomal miRNA shows differential expression of miRNAs upregulated in patients with malignant ascites when compared to benign ascites

**Figure 5:**
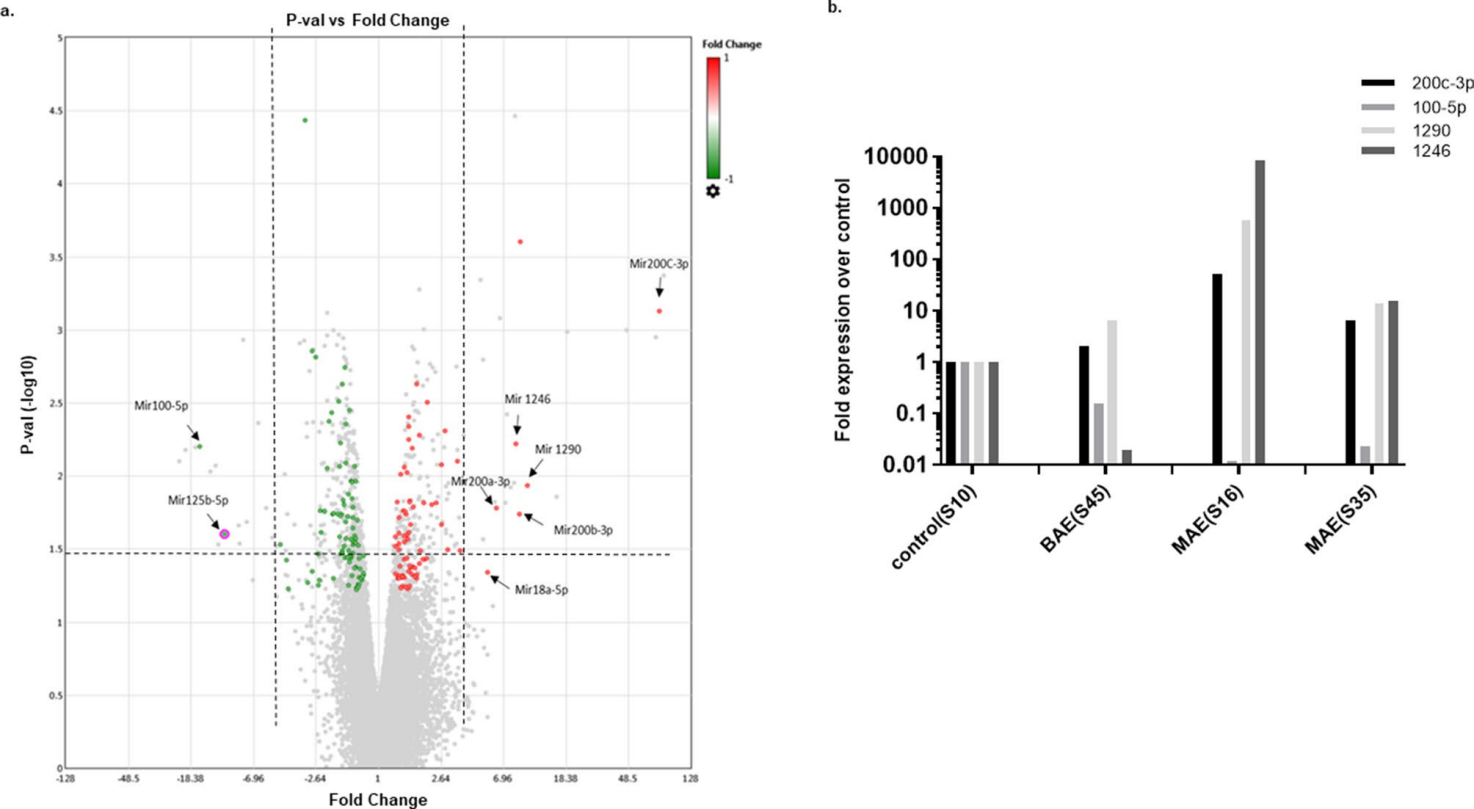
Quantitative Real-time PCR validation of miRNA in exosomes derived from ascitic supernatant. miRNA profiling was done using RNA isolated from BAE (benign ascitic supernatant derived exosomes) or MAE (malignant ascitic supernatant derived exosomes). P-value vs. fold change is depicted as a volcano plot generated using TAC software (ThermoFisher) (**a**). Volcano plot of significant expression of miR 200c-3p, miR 100-5p, miR 1290, and, miR 1246 over control in BAE (S45), and MAE (S16 and S35) (**b**).

Exosomal RNA was isolated from both benign and malignant ascites derived EVs and sent to the core lab for Affymetrix miRNA expression Profiling. miRNAs, which were above five-fold upregulated or down-regulated with a p ≤ 0.06 in malignant as compared to benign EVs, were considered as shown in the volcano plot (Table 1 and Fig. 5a). The quality of exosomal RNA was analyzed using a 2100 bioanalyzer (Agilent) (Supplementary Figure S2), and real-time PCR was performed to validate differentially abundant miRNA. miR200c-3p, miR18, miR1246, miR1290 were expressed at higher levels in malignant ascites while miR100-5p, miR125b-3p had lower expression malignant ascitic fluid derived EVs as compared to the benign ascitic EVs (Fig. 5a,b).

### Gene expression array demonstrates differential expression of genes in tumor cells engrafted in exosome treated omentum

**Figure 6:**
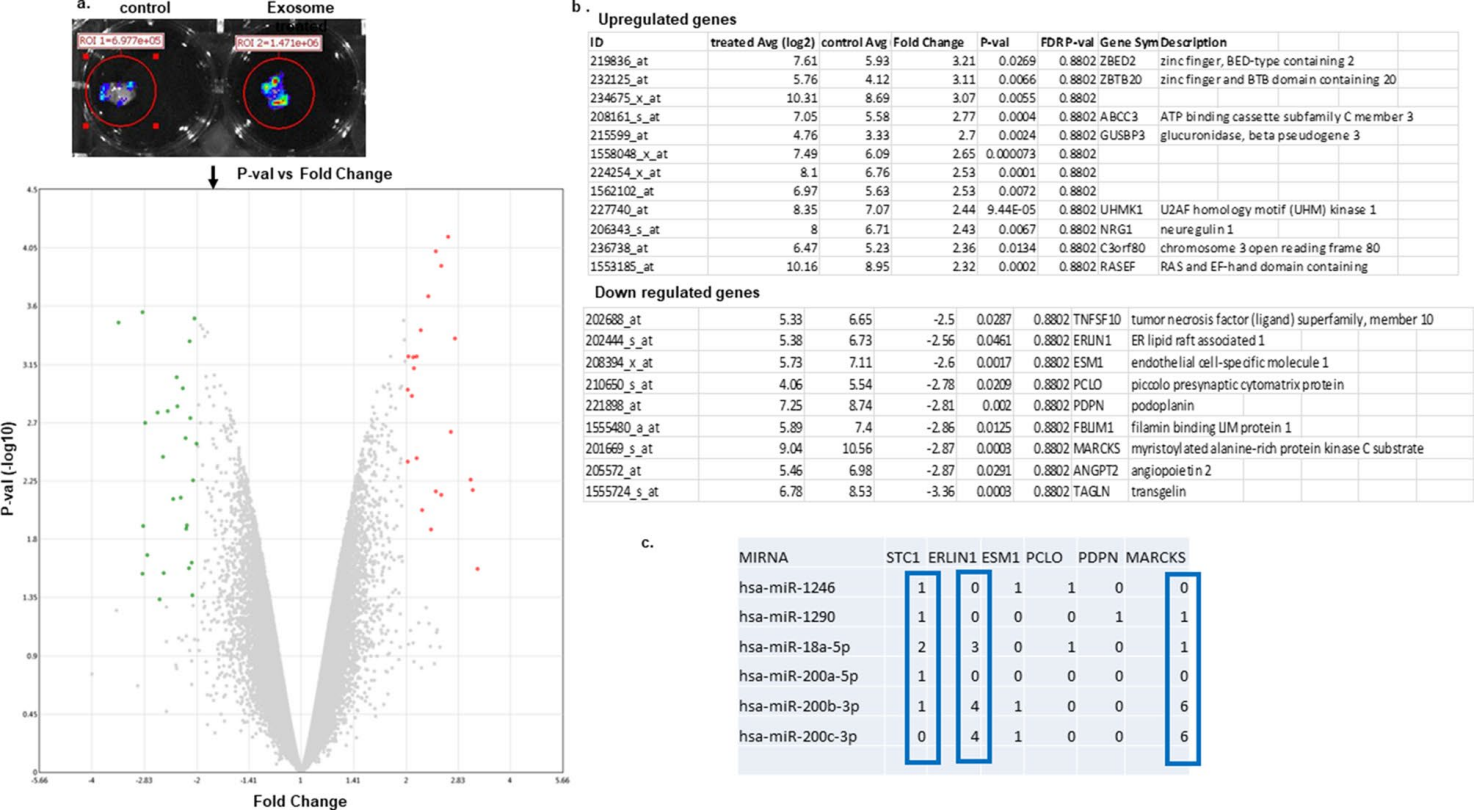
Ex vivo migration assay. Luciferase labeled SKOV3cells were allowed to migrate towards pieces of fresh omentum, which are either untreated (control) or treated with 500 μg exosomes for 5 days. IVIS imaging shows the increased amount of cells migrated to treated omentum (**a**). The GFP labeled cells were isolated from omentum were allowed to grow, and gene expression assays performed. The upregulated and downregulated genes with two-fold change are shown (**b**).  A list of miRNAs predicted to bind to the downregulated genes in the cells migrated towards omentum pretreated with malignant ascitic derived EVs (**c**).

We performed a gene expression microarray analysis with RNA isolated from cells migrating towards patient derived omentum tissue alone or omentum pretreated with EVs isolated from ascitic supernatant of high-grade ovarian cancer patients (Fig. 6a,b). We found that UHMK1, ABCC3, RASEF1, NFAT5, ZBTB20 were at least two-fold upregulate, while MARCKS, TGCN1, ROR1, STC1, ERLIN1, IFITM1 were at least two-fold downregulated in cells migrating towards malignant exosome treated omentum as compared to untreated omentum (a p-value provided in Fig. 6b). To determine if exosomal miRNA in malignant EVs would be predicted to bind to mRNA of the genes that were found to be downregulated in tumor cells after treatment with EVs, we used the miRsystem database (http://miRsystem.cgm.ntu.edu.tw), which has seven miRNA target identifying programs integrated (Target Scan, miRanda, PicTar, PITA, rna22, miRBridge, and DIANA). A list of miRNAs predicted to bind to the mRNA of the genes that were downregulated in the cells that migrated towards omentum pretreated with malignant ascitic derived EVs was generated in Fig. 6c.

## Discussion

Malignant ascites is a unique tumor microenvironment that supports the growth and dissemination of ascites spheroids. This fluid contains extracellular vesicles that may contribute to the survival, chemoresistance, and metastasis of these ascites spheroids^8^. The exact stoichiometry of EVs required to their effects is unknown. Once internalized, the miRNAs can target various molecules related to the growth and spread of tumors and also affect cancer signaling pathways^9^. We found that EVs derived from malignant ascitic fluid increased ovarian cancer cell growth, migration, motility, expansion in a 3D matrix as well as spheroidal growth. (Supplementary Figure S1). The malignant ascitic fluid derived EVs treated with RNAse failed to form as many spheroids when compared to the untreated ones, thus pointing towards the role of EVs as miRNA carriers.

miRNA profiling of the exosomal RNA revealed increased expression of miR-200a/b/c, miR-1290, miR-1246, miR-18b-5p, and a down-regulation of miR-100-5p and miR125b-5p in malignant ascitic EVs as compared to benign EVs. These same miRNAs were reported by Yang et al. in ovarian cancer as compared to normal ovary; miR 100, and miR125b was ninefold, and fivefold downregulated, and miR 200a was 5.75 fold upregulated^10^. miR-100 belongs to the MIR100HG host gene cluster, which also produces miR-125b and let7A2 and maps to chromosome 11q24.1. It has been reported that the expression of miR-100 and miR-125b are downregulated in different histotypes of ovarian carcinomas as compared to normal ovarian tissue^11^. miR-200 and miR-18 are found to be upregulated in ovarian cancer^12^. We found the addition of EVs derived from patient S16 ascites transfected with miR-100 mimics reduced the number of ovarian cancer spheroids as compared to EVs transfected with control mimics (Supplementary Figure S3).

We identified MiR-1290 and 1246 as upregulated in EVs from malignant ascites as compared to those from benign. Recently exosomal miR-1246 has been reported as a serum biomarker for breast cancer by targeting cyclin G2 and also found to induce motility and invasion of oral squamous carcinoma^13^. Both of these miRNAs have been found to be upregulated in tumors as compared to their normal tissues of breast, colon, head, and neck. The role of miR-1246 and miR-1290 as CD166 positive tumor-initiating cell derived miRNAs in lung cancer contributing to therapy response has been highlighted^14^. Furthermore, miR-1290 has been shown to activate the wnt pathway and increase nanog and c-myc in colon cancer implying its probable contribution to stemness^15^. Since both miR-1246 and miR-1290 are upregulated in EVs in malignant ascites, it's possible that they may have contributory effects on increasing the cancer-initiating capacity of ovarian cancer cells.

The migration of ovarian spheroids to omental tissue reflects their metastatic capabilities. Ovarian cancer spheroids grown with EVs isolated from ascitic fluids of patients diagnosed with high serous malignant carcinoma listed in Table 2 showed increased growth potential and enhanced migratory properties both in vitro and ex vivo towards patient-derived omental tissues. We further isolated cells migrated to omentum either pretreated with malignant ascites derived EVs or to untreated omentum (control) and performed gene expression analysis. Our results show that ABCC3, ZBED2, ZBTB20, NFAT5, and UHMK1 were upregulated in cells that migrated into treated omentum as compared to those that migrated into the untreated omentum, whereas MARCKS, Angiopoietin, and Transgelin were downregulated. ABCC3 is a well-known drug transporter that transports fewer anticancer drugs than others and has been reported to be resistant to epipodophyllotoxin, inhibitors of topoisomerases such as etoposide and teniposide, cefadroxil and fexofenadine^16, 17^. We treated ovarian cancer cells with EVs derived from benign and malignant ascites and subjected them to drug sensitivity assays. We found that the cells treated with EVs from malignant ascites were resistant to etoposide treatment but not significantly sensitive to carboplatin or paclitaxel (Supplementary Figure S4). ZBTB20 and UHMK1 are both cell cycle regulators that upregulate cyclin D1 and cyclin E and regulate p27 expression^18–20^. UHMK1or kinase interacting with stathmin has been reported to be a mediator of signaling from secretory RNA transporting granules to the nucleus^21, 22^. Among the downregulated genes, MARCKS is associated with bortezomib resistance by regulating p27 pathway^23, 24^. It is associated with exocytosis of ubiquitin associated vesicles to alleviate proteolytic stress^25^. EVs contain miRNAs, proteins, and lipids, which may influence cell behavior. We treated the EVs with RNAse or Trypsin and assessed their influence on ovarian cancer spheroid growth or migration to omental fat. We found that RNase treated EVs abrogated migration of ovarian cancer cells towards omental tissue and also reduced the size and number of ovarian cancer spheroids in vitro. We queried if any of the upregulated miRNAs in malignant ascites derived EVs would target the genes that were down regulated in cells that migrated to omental fat tissue. We found that miR 200c-3p, miR18a, and miR1290 are predicted to target MARCKS, ERLIN1and also stanniocalcin1. MARCKS silencing has been shown to increase UHMK1 expression and proliferation of endothelial cells^26^. UHMK1which is reported to be a direct transcriptional target of YAP and FOXM1 can support cell proliferation by inducing cell cycle regulators and destabilizing cytoplasmic tubulin^27, 28^. YAP is known to be an active transcriptional cofactor involved in Hippo and Notch signaling pathways. Its sensory role in providing cues for three-dimensional growth by estimating the geometry and physicality of the microenvironment is well reported^29^. DLK1, a non-canonical notch ligand, is highly expressed in High grade serous ovarian carcinoma and has been associated with poor overall survival and PFS^30^. We find increased expression of UHMK1 in cells isolated from omental fat treated with malignant ascitic EVs, which may explain the enhanced migratory capacities of these cells.

Our studies highlight the role of EVs isolated from ascitic fluid of high grade serous ovarian cancer patients in enhancing the growth potential of ovarian cancer spheroids. EVs showed high expression of miRNAs 1290, 1246, and 200c and low expression of miR 100-5p, all of which have reportedly been shown to be involved in autophagy of ovarian cancer. The EVs also increased the migration of ovarian cancer cells towards omental fat, suggesting they play a critical role in this process. In total, our results suggest that targeting extravesicular miRNA signaling in ascites may be a useful strategy to inhibit the process of omental metastasis that is a frequent process for women with advanced ovarian cancers.

## Materials and methods

### Patient samples

Patients were taken to the operating room by the gynecologic oncology service at the University of Texas, M.D. Anderson Cancer Center, with a suspected diagnosis of ovarian cancer. Patients were enrolled on an IRB approved protocol. Informed consent was obtained to collect ascites fluid at the time of a diagnostic laparoscopy or cytoreductive surgery. All samples were acquired prior to receiving any treatment. Collected ascites fluid was centrifuged at 1200 rpm for 10 min to pellet cells. The upper supernatant fluid layer was collected for EVs isolation. The remaining pellet was resuspended in RPMI 1640 with L-glutamine (MediaTech) and then transferred over Ficoll-Paque Plus Media (GE Healthcare) for gradient separation. The resulting ascites cell buffy coat was carefully removed. After washing with a series of centrifuge steps and fresh RPMI 1640 media, isolated ascites cells were utilized for the spheroid growth assay. Clinical characteristics information was extracted from the medical record (Table 2). Two patients taken for surgical staging of suspected ovarian cancer (Table 2, study no. S10, no. S45) were instead diagnosed with benign disease, Meig's syndrome or borderline tumor. The number of benign patients is a limitation as this is a rare condition but provided a valuable comparison to the malignant ascites samples.

### Cell culture

Human ovarian cancer cell lines, OVCA433 and OVCA429, generously provided by Dr. Samuel Mok (University of Texas, M.D. Anderson Cancer Center), were grown in DMEM containing 10% FBS with 1% Penicillin–Streptomycin antibiotics. HeyA8 cells, generously provided by Dr. Deepak Nagrath (Rice University), were grown with MEM containing 15% FBS. SKOV3 cells, generously provided by Dr. Michael Andreeff (University of Texas, M.D. Anderson Cancer Center), were grown in McCoy's media with 10% FBS. SKOV3 cells stably transduced with a red fluorescent protein and renilla luciferase (rLuc) using lentivirus was grown in DMEM with10% FBS^31^.

### Spheroid growth assay

Ovarian cancer spheroids were cultured as described before^32^. Briefly serum-free MEM supplemented with 20 ng/ml human recombinant epidermal growth factor (EGF; Invitrogen), 10 ng/ml basic fibroblast growth factor (BFGF; Invitrogen), and B27 was used to grow spheroids in six well ultra-low attachment plates.

### Isolation of EVs

2 ml of ascitic supernatants previously collected from chemo naive patients were thawed out on ice and passed through 0.2 μm filters. EVs were isolated using Exoquick (System Biosciences) as per manufacturer's instructions^33^. The concentration of EVs was calculated by taking A2780 measurements on a Nanodrop. The EVs were diluted to 1 mg/ml and then used at required concentrations (250 μg/ml) for assays.

### Electron microscopy

Electron micrscopy was carried out following the protocol described by Shi et al*.*^34^. Samples were placed on 100 mesh carbon coated, formvar coated copper grids treated with poly-l-lysine for approximately 1 h. Samples were then negatively stained with Millipore-filtered aqueous 1% uranyl acetate for 1 min. The stain was blotted dry from the grids with filter paper, and samples were allowed to dry. Samples were then examined in a JEM 1010 transmission electron microscope (JEOL, USA, Inc., Peabody, MA) at an accelerating voltage 80 kV. Digital images were obtained using the AMT Imaging System (Advanced Microscopy Techniques Corp., Danvers, MA).

### Western blotting

Western Blotting was performed according to the manufacturer's protocol provided by EXOAB-Kit (System Biosciences, Palo Alto, CA, USA). In brief, EVs were lysed in RIPA lysis buffer (25 mM Tris–HCl pH 7.6 150 mM NaCl 1% NP-40 1% sodium deoxycholate 0.1% SDS). Proteins (20 μg) were resolved by 10% SDS-PAGE and immunoblotted on PVDF membranes. The membranes were incubated with primary antibodies for Alix (1:1000, Cell Signaling Technology, Danvers, MA, USA) or rabbit anti-human CD63, CD9, and CD81 (EXOAB-Kit) (1,1:1000) overnight at 4 °C, washed with Tris Buffered Saline with 0.05% Tween. The membranes were then incubated with goat anti-rabbit HRP secondary antibodies (1:20,000). Blots were developed using Super Signal (Pierce, Rockford, IL, USA).

Real-Time PCR: EVs were isolated from 1 ml ascitic supernatant and then isolated miRNA using the ExoRNAeasy from QIAGEN. An equal amount of starting miRNA was used to generate cDNA. cDNA was synthesized using a Fast cDNA synthesis kit from ABI ThermoFisher. Real-time PCR was performed on the ABI 7500 Fast PCR using TaqMan Fast Advanced miRNA assays as per manufacturer’s protocol 478,224 hsa-mir-100-5P; 477,881 hsa-mir-1246; 477,895 hsa-mir-1290; 478,351 hsa-mir-200c-3p; 477,940 hsa-mir-186-5p.

### miRNA profiling

The miRNA gene expression profiling was performed on Affymetrix Genechip miRNA 4.0 array according to the protocol by Dee and Getts^35^. In brief, 600 ng of total RNA samples were labeled with the FlashTag Biotin RNA Labeling Kit (Flash tag kit, Affymetrix, Santa Clara, CA). The labeled RNA was quantified and hybridized onto the miRNA 4.0 microarray according to the standard procedures provided by the manufacturer. The labeled RNA was heated to 99 °C for 5 min and then incubated at 45 °C for 5 min. RNA-array hybridization was performed with agitation at 60 rotations per minute (RPM) for 16–18 h at 48 °C on an Affymetrix F450 Fluidics Station (Affymetrix, Santa Clara, CA). The chips were washed and stained using a Genechip Fluidics Station 450 (Affymetrix). The chips were then scanned with an Affymetrix GeneChip Scanner 3000 (Affymetrix). Signal values were computed using the Affymetrix GeneChip Command Console software (Affymetrix). Data analysis was done using TAC software (Applied Biosystems).

### Migration assay

Migration assays were conducted using 8-μm polyethylene terephthalate filters (BD). Cells (treated with EVs or control), which migrated to the lower sides of the trans-well, were stained using 0.05% crystal violet, and the cell number was counted. Each test group was assayed in triplicate. Four different fields of each insert were photographed at 10 × magnification. Each field was divided into quadrants, and cells in diagonally opposite quadrants were counted^36^.

### Ex vivo migration assay

Fresh pieces of omentum adipose tissue measuring 25 mg was cut and placed into 24 well plate with 300 μl of 3D media without B27 supplement in a low attachment plate. 500 μg of EVs were added to the omentum. Then SKOV3 (rLuc) cells were plated at a density of 20,000cells/ well. Coelenterazine (Biotium, CA, USA) working solution was added to a 24 well plate, and the tissue pieces transferred and incubated at 37 °C for 1 h. Luminescence was measured with Xenogen IVIS bioluminescence/fluorescence optical imaging system (Caliper Life Sciences) at day5. The omental migration assays were repeated twice with two BAEs and two MAEs. All samples were used in triplicate.

### Scratch assay

Scratch assay was done as described elsewhere^36^. Briefly, 500,000 cells were plated for scratch assay. A central linear scratch was made with pipet tip in confluent six well plates where cells were grown either in the presence of 250 μg/ml exosomes or control. Pictures of wound cultures were taken at 0 and 16 h (average doubling time is 18–20 h). The results are expressed relative to control cell migration. Each test group was assayed in triplicate, and the results are expressed relative to control cell migration.

### Colony forming unit assay

500 cells were plated into each well of a six-well plate in media with 250 μg/ml EVs along with control cells (without EVs), allowed to grow for 10 days, and stained with crystal violet. Six different fields were counted for the number of foci, and data are represented as average ± S.E.^37^.

### Microarray analysis

We isolated cells that migrated to omentum tissue, which was either treated with EVs or not treated with EVs. RNA was isolated from both sets and sent to MD Anderson core lab for Gene microarray. Affymetrix Gene microarray was performed as per manufacturer's instructions. More than two-fold upregulated or downregulated genes in cells isolated from omentum treated with exosomes or from untreated omentum tissue are included in the analysis.

### RNase treatment

Evs were treated with 100 μg/ml RNase A (Thermo Fisher Scientific) for 20 min at 37 °C followed by incubation with RiboLock RNase Inhibitor (Thermo Fisher Scientific).

### MTT assay

Cells with or without EVs were plated in 96 well tissue culture treated plates at a density of 5 × 10^3^ cells per well. The cells were incubated for 72 h, and cell viability was assessed using MTS assay (Promega).

### Three dimensional culture

3D cultures were grown following the protocol by Debnath et al*.*^38^. Briefly, chambered coverglass slides were coated with a phenol-free 3D culture matrix from Trevigen (Gaithersburg, MD.USA). Five thousand cells were mixed with a 2% culture matrix and plated in every well. EVs were added at a concentration of 250ug/ml, and culture maintained for 7 days.

### EVs with miRNA100 mimics

Lipofectamine 2000 were used to transfect miRNA 100 mimic into MAE from sample no. S16 (control). The loaded miRNAs were then added to cells that were cultured as spheroids.

### Statistical analysis

Most assays (spheroid growth, microarray) have been performed with all seven patients and in vitro assays repeated three times with different passages of cell lines. Data sets were analyzed using the Graph Pad Prism 8.00 software for Windows (Graph Pad Software, La Jolla, CA, USA). One-way ANOVA followed by Tukey's post test was used for multiple comparison analyses. *P* < 0.05 were considered significant.

### Ethics approval

All methods were performed under protocol 2019-0952, approved by the Institutional Review Board of the University of Texas, M.D. Anderson Cancer Center.

## Supplementary Information

Supplementary information.
