# Supplementary material for: Extracellular vesicles derived from ascitic fluid enhance growth and migration of ovarian cancer cells
Source: Sci Rep. 2021 Apr 28;11:9149. doi: 10.1038/s41598-021-88163-1 (PMC8080718; doi:10.1038/s41598-021-88163-1)
Supplement: Supplementary file 1 — Supplementary information. [file 41598_2021_88163_MOESM1_ESM.pdf]

Extracellular Vesicles Derived from Ascitic Fluid Enhance Growth and Migration of Ovarian Cancer Cells

Aparna Mitra[1], Kyoko Court[1], Travis Solley[1,] Megan Mikkelson[1], Chi Lam Au Yeung[2], Alpa Nick[3], Karen Lu[1] and Ann H. Klopp[1]*

1 Department of Radiation Oncology, The University of Texas MD Anderson Cancer Center, 1515 Holcombe Boulevard, Houston, TX 77030, USA.

Electronic address: aklopp@mdanderson.org.

2Department of Gynecologic Oncology and Reproductive Medicine, The University of Texas MD Anderson Cancer Center, Houston, TX 77030, USA.

3 Tennessee Oncology, Nashville, Tennessee 37203, USA.

Supplemental I:

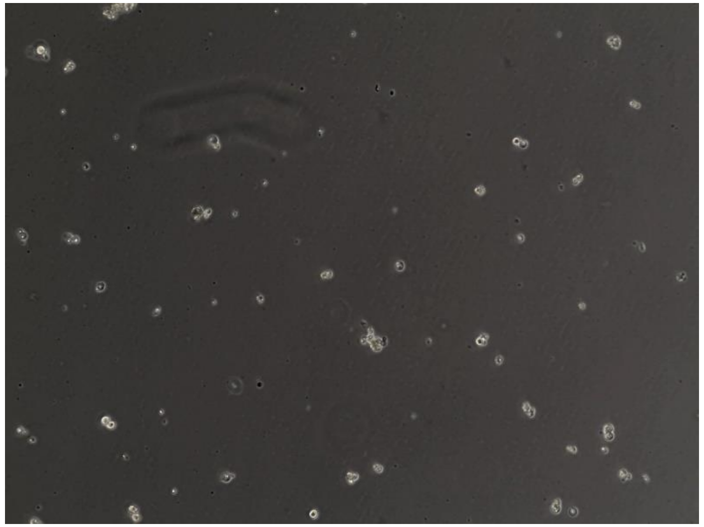

Malignant Ascites S35

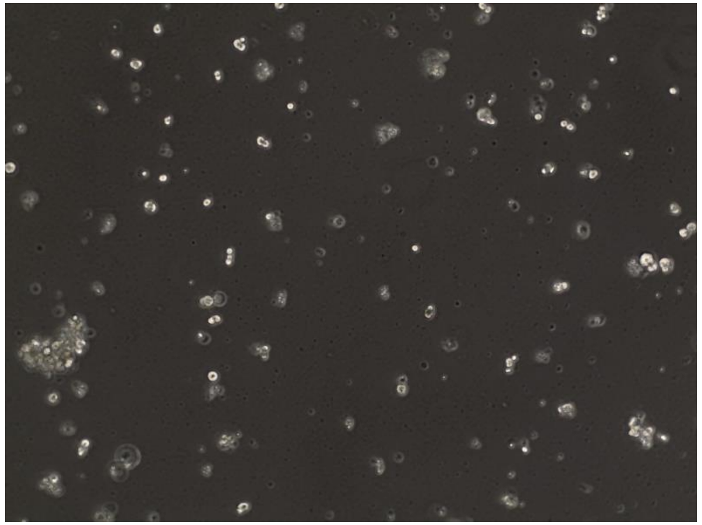

Malignant Ascites S35 + Benign exosome (S10)

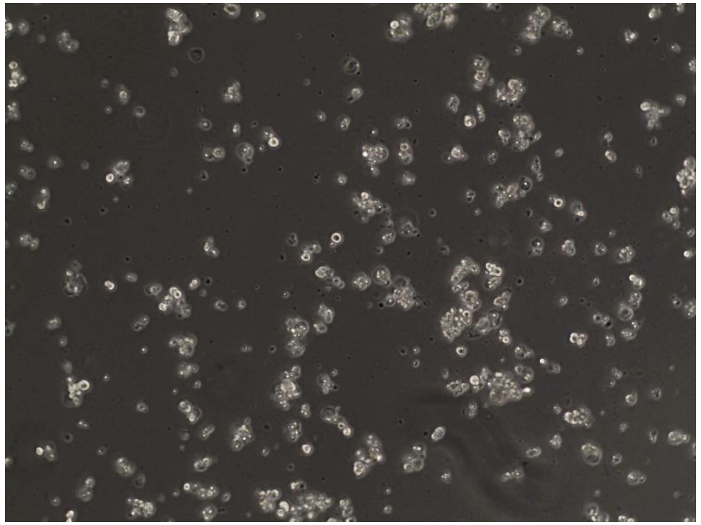

Malignant Ascites S35 + Malignant exosome (S16)

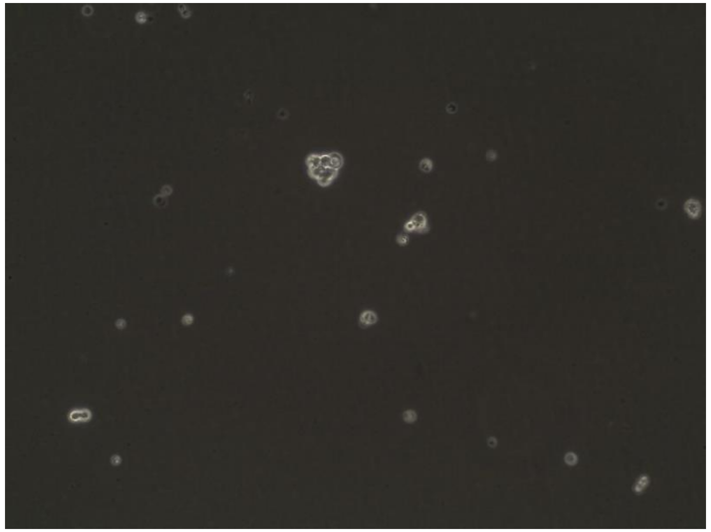

Malignant Ascites S16

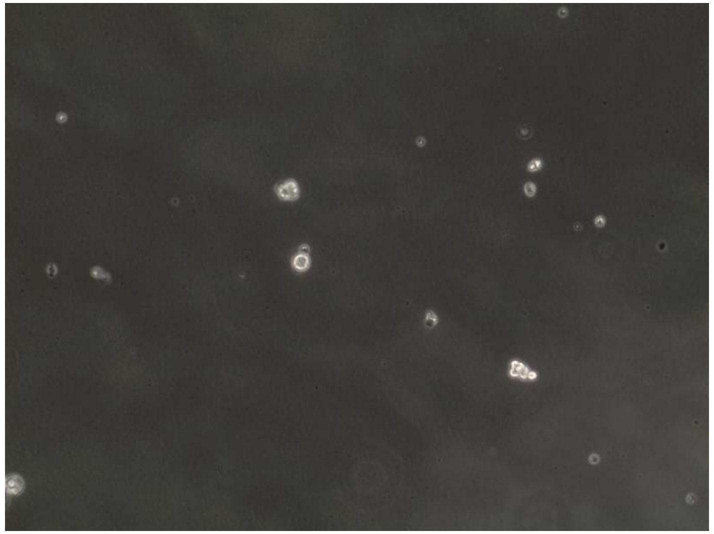

Malignant Ascites S16 + Malignant exosome (S35)

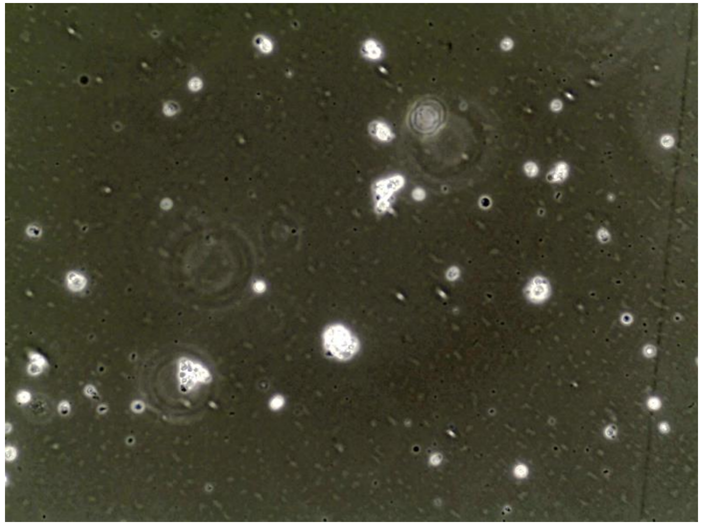

Malignant Ascites S16 + Malignant exosome (S35)

Supplemental II:

No peaks detected at 28S and 18S positions of the exosomal miRNas

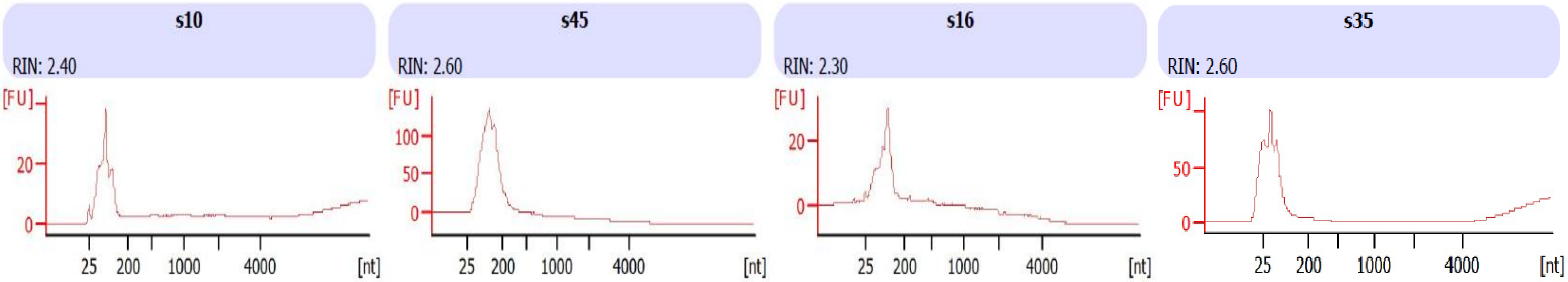

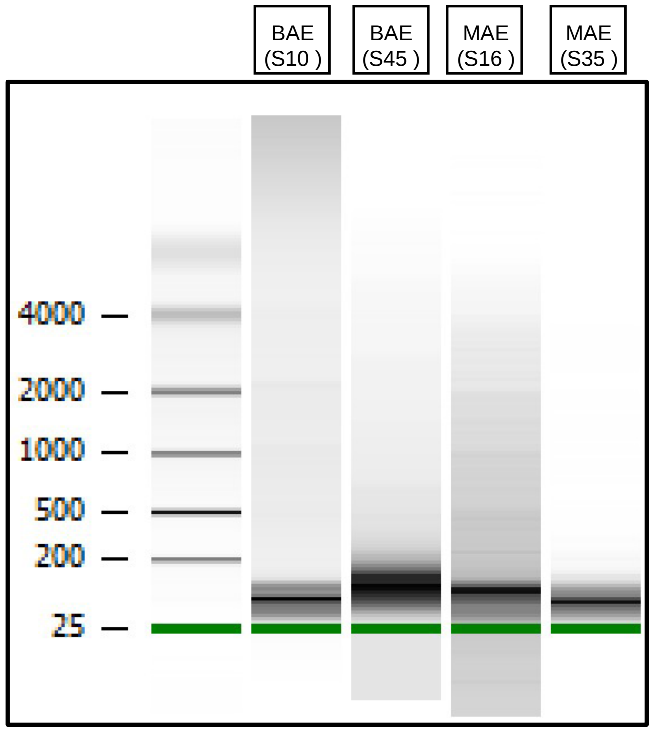

Supplemental III:

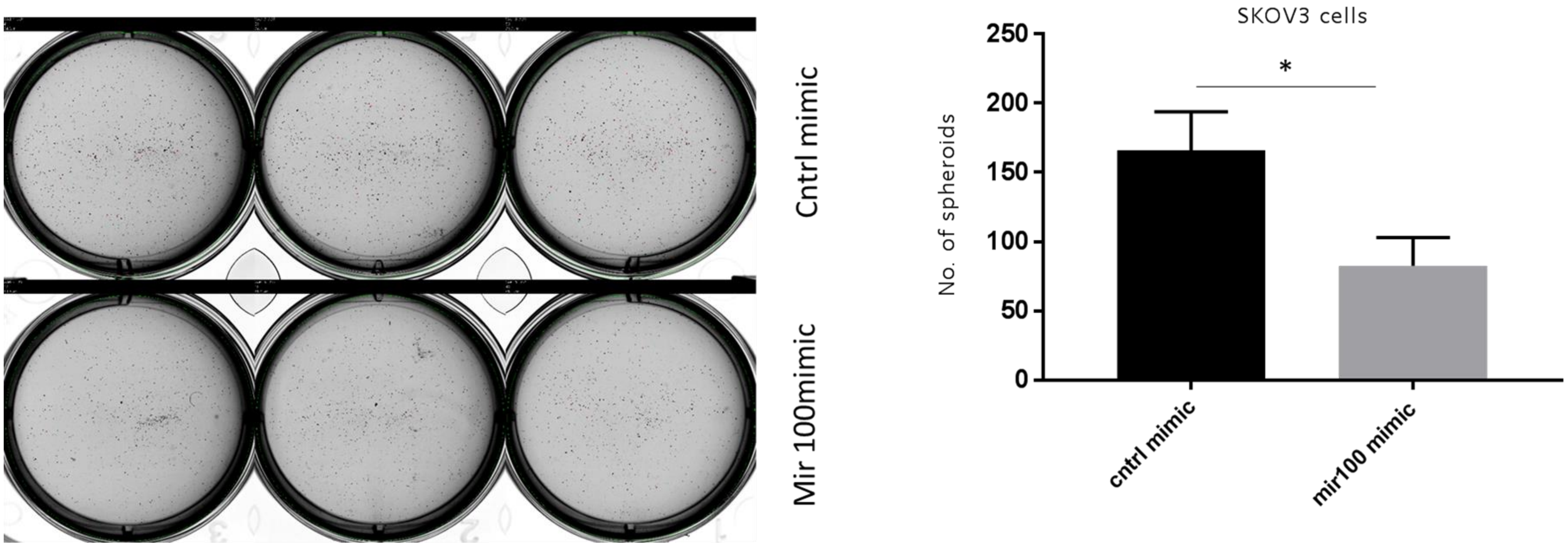

Supplemental V:

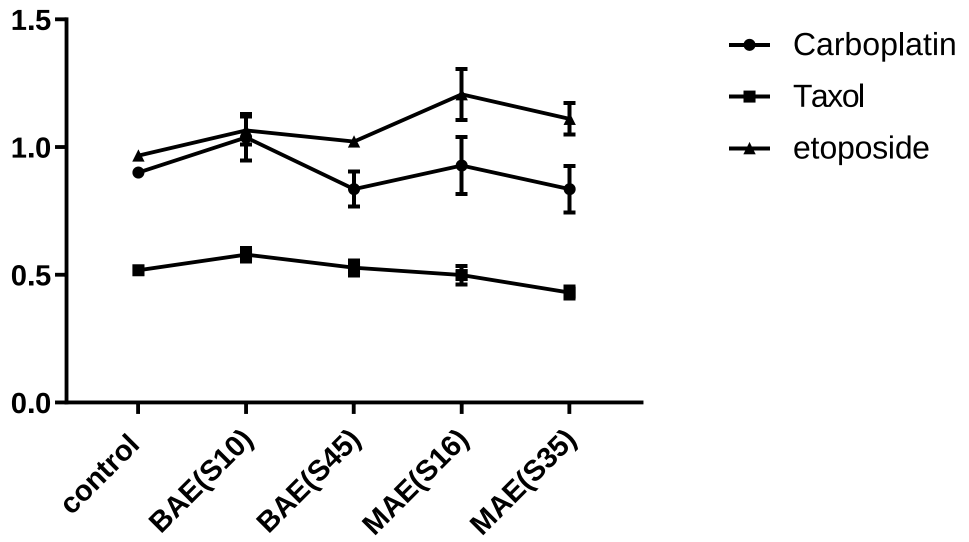

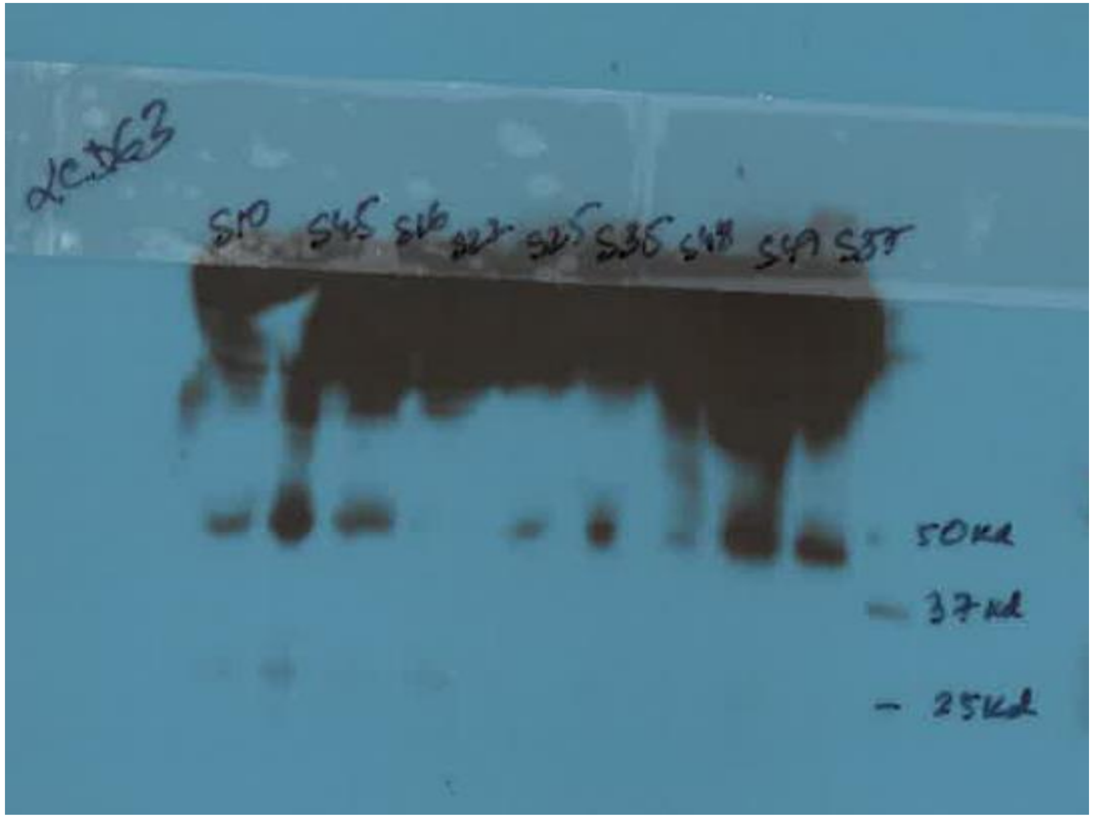

Anti-CD 63, Non reducing

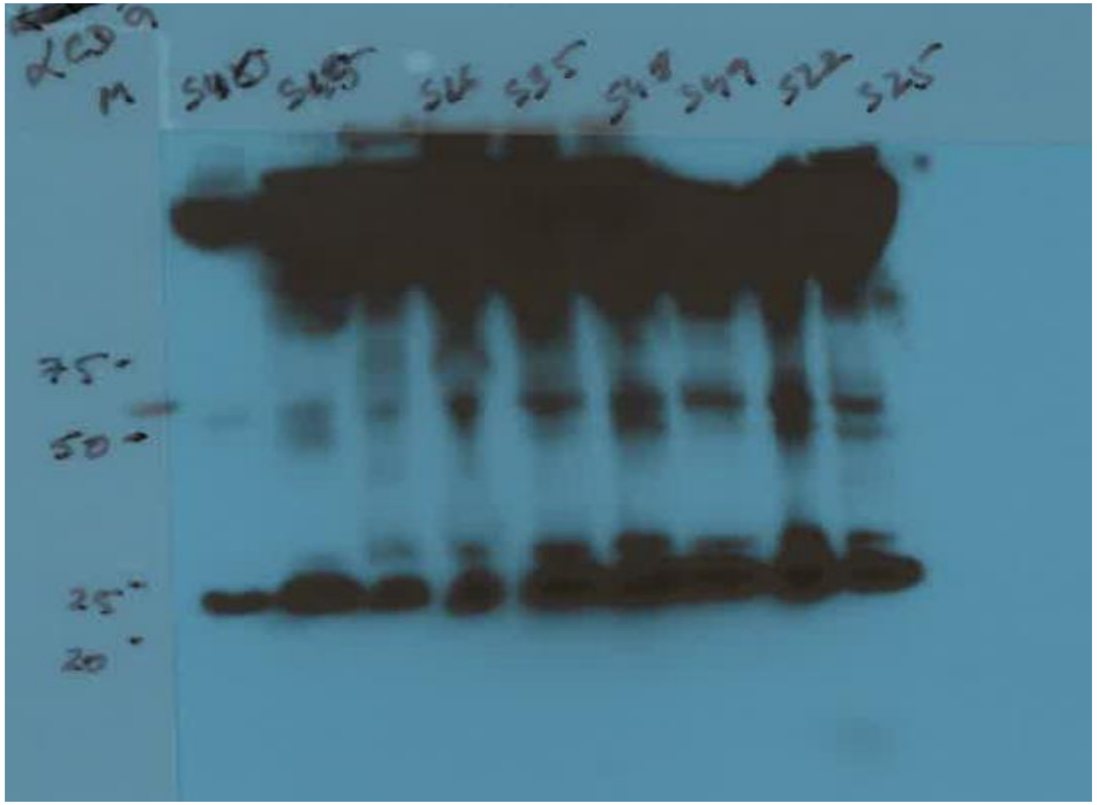

Anti-CD 9, Non reducing

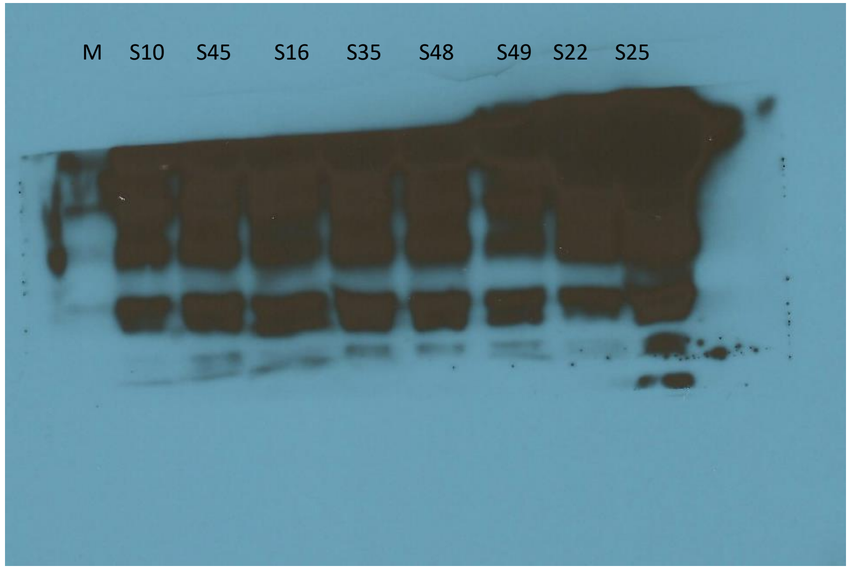

Anti-CD 81, Non reducing

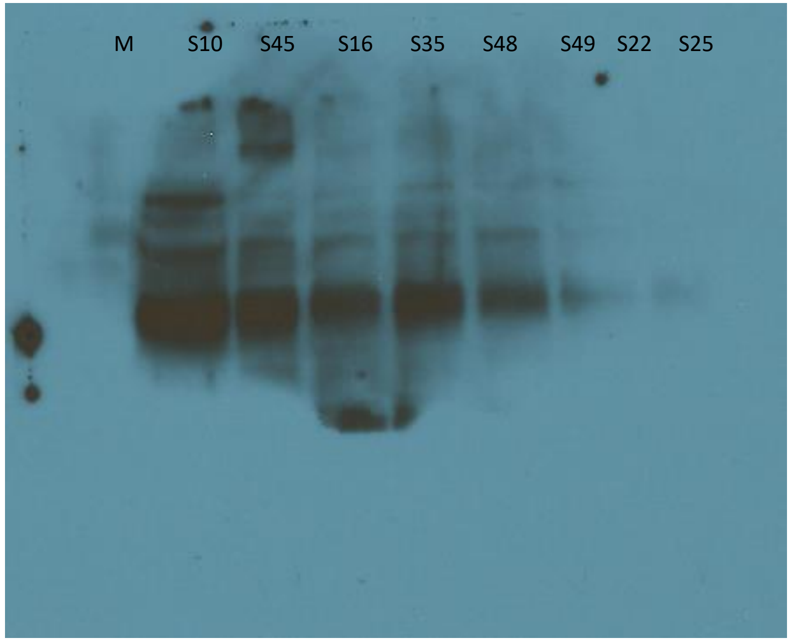

Anti-Alix , high exposure

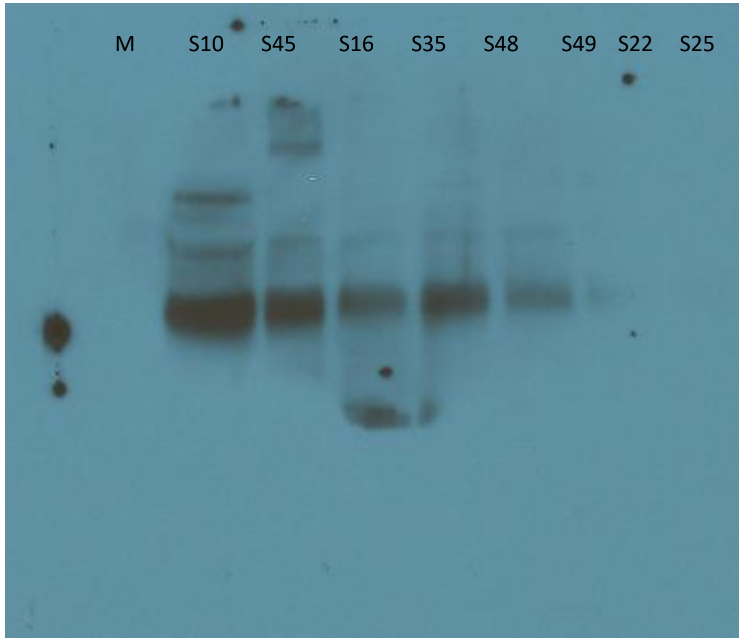

Anti-Alix , low exposure
